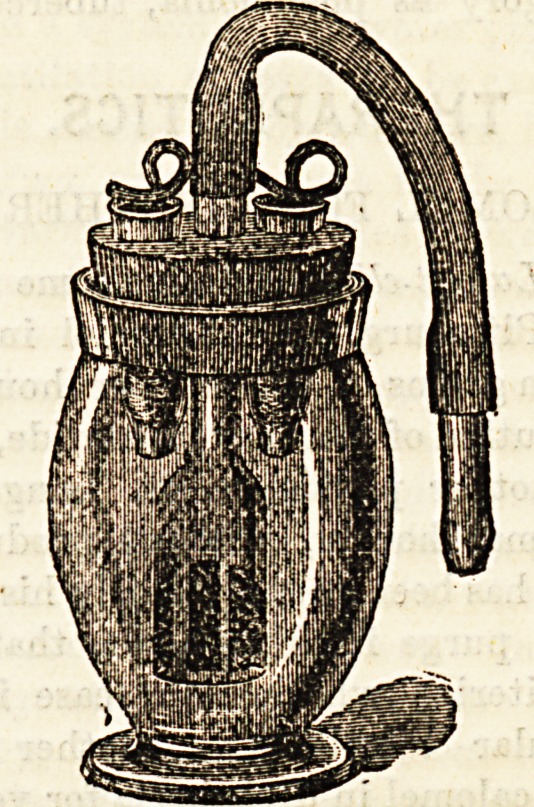# New Drugs, Appliances, and Things Medical

**Published:** 1890-04-05

**Authors:** 


### NEW DRUGS, APPLIANCES, AND THINGS MEDICAL.

[All preparations, appliances, novelties, etc., of which a notice is desired, should be sent to The Editor, The Lodge, Porchester Square, W.]

### THE HORTON ICE-CREAM COMPANY, 56, GRAY'S INN ROAD.

The above have sent us some samples of their patent beef tea (Bovril) ice. The idea of always having iced restoratives ready is a good one, and we do not doubt that the company's preparations will be found useful in those cases of persistent vomiting and the like, where only minute quantities of food can be retained by the stomach. We would suggest that other forms of soup should be kept in the frozen state, so that patients may have a choice. We have found sometimes that a change in the flavour of the nourishment has done great good in the way of its retention. We shall make a note of the firm's address, as freezing soup is a difficult art possessed by few cooks.

### SANTHA.

This is an extract of tea in which the tannin is combined and neutralized. It preserves the aroma and flavour of tea better than any form of tea extract with which we are acquainted. The fact that the tannin is in a combined state should enable some of those who cannot enjoy their favourite drink in the ordinary form of infusion, to be able again to indulge in "the cup that cheers," and bless the Darjeeling Tea Company, who have introduced it.

### GODFREY'S CHLORIDE OF AMMONIUM INHALER.

We have received from Messrs. Godfrey and Cook, Conduit Street, a specimen of the above, of which we here give an illustration. Having tried many varieties of chloride of ammonium inhalers, and found them impracticable because of the complexity of their bottles and cross tubes, we one day obtained a G. and C.'s inhaler. We have always used them since. They are at once safe, simple, and self contained, and having once demonstrated to your patient the drill for combining the active ingredients, only the densest minds cannot make a mistake afterwards. The directions are as follow: Take out the cork, moisten the sponge in the large tube, put back the cork. Dip the green holder in the green bottle (hydrochloric acid) and put it into the green tube. Dip the white holder in the white bottle (dilute ammonia) and put it into the white tube. Having done this, the apparatus is ready for use. Combinations of the vapour with pine oil, eucalyptus, and the like, can easily be made by dropping the desired agent on to the sponge. The virtues of chloride of ammonium vapour are too well known to require enumeration, but we can state that we have found it of great service in the prolonged sore throats following an attack of influenza, as well as in the bronchial conditions produced by that disease. One word in warning. Hydrochloric acid is corrosive; tne immediate addition of an excess of the ammonia solution to spots produced by the acid on cloth, &c., will restore the normal colour and neutralize the acid.

### SALT REGAL, PATENTED.

A pleasant form of effervescing saline, remarkable for containing a small portion of a permanganate salt. This, on addition of water, causes a pink colour to be developed, and this identifies the preparation from the countless other popular forms of a similar nature. The whole idea is ingenious, the mixture being palatable and pleasant, and so likely to prove generally appreciated.

## Figures and Tables

**Figure f1:**